# Caregiver burden in schizophrenia following paliperidone palmitate long acting injectables treatment: pooled analysis of two double-blind randomized phase three studies

**DOI:** 10.1038/s41537-017-0025-5

**Published:** 2017-07-27

**Authors:** Srihari Gopal, Haiyan Xu, Kelly McQuarrie, Adam Savitz, Isaac Nuamah, Kimberly Woodruff, Maju Mathews

**Affiliations:** 1 Janssen Research & Development, LLC, Raritan, NJ USA; 2 Janssen Scientific Affairs, LLC, Raritan, NJ USA

## Abstract

The pooled analysis of two double-blind, randomized, multicenter, phase-3 studies evaluated predictors of improvement or worsening of schizophrenia-related caregiver burden following paliperidone palmitate long-acting injectables (1-monthly [PP1M] and 3-monthly [PP3M]) treatment. Caregivers were offered to complete the involvement evaluation questionnaire (involvement evaluation questionnaire; 31-item scale). Total, 1498 caregivers (intent-to-treat open-label analysis set, *n* = 1497; mean [SD] age: 51.5 [13.02] years, 27 countries) were included: 49% were parents and >50% caregivers spent >32 hours/week in caregiving. Majority of caregivers with considerable burden (*n* = 1405; mean [SD] baseline involvement evaluation questionnaire scores: 28.4 [15.07]) improved significantly from baseline to end-of-study (*n* = 756; mean [SD] change from open-label baseline to double-blind endpoint in long-acting injectable scores:−8.9 [14.73]); most improvements were seen in urging followed by worrying, tension, and supervision domains (mean [SD] change from open-label baseline to double-blind endpoint in involvement evaluation questionnaire scores, urging: −3.7 [6.45]; worrying:−2.6 [5.11]; tension:−2.3 [4.84]; supervision: −1.3 [3.69]). Improvements significantly correlated with relapse status, patient age, and age of diagnosis (*p* < 0.001) while long-acting injectable use at baseline, number, and duration of prior psychiatric hospitalizations (<24 months) had no significant correlation. Caregiver burden was significantly improved for patients on prior oral antipsychotics post-switching to long-acting injectable, with less impact on leisure days and hours spent in caregiving (*p* < 0.001). Family members of patients with schizophrenia experience considerable caregiver burden. Switching from oral antipsychotic to long-acting injectable can provide meaningful and significant improvement in caregiver burden.

## Drug formulation: Easing the toll of caregiving

Switching from oral to long-acting injectable antipsychotic medication improves overall caregiver burden. The physical, emotional and financial toll of providing care for patients with schizophrenia is often underestimated. Poor adherence to conventional oral antipsychotics is a major cause of symptomatic relapse in patients and of stress for carers. Srihari Gopal and colleagues at Janssen Pharmaceuticals have pooled data from two large studies involving 1498 caregivers across 27 countries. They found that administration of either 1- or 3-monthly long-acting injectable antipsychotics not only eased the burden of daily dosing and patient compliance, but also had a positive impact on the stress conditions of caregivers. Using the Involvement Evaluation Questionnaire to measure caregiver burden, the authors showed that the switch in drug formulation decreased the need to urge patients to self-care and the hours spent caregiving.

## Introduction

Caregiver burden in schizophrenia is significant, though often underestimated.^1^ Studies demonstrated that in western countries, about 25–50% patients with schizophrenia live with their caregivers and depend on their assistance. In Asian countries, patient dependency on caregivers is as high as 70%.^2^ This burden causes increased work load, sleep disturbance, financial problems, and decreased leisure hours for caregivers.^1^ A systematic review for the global population with schizophrenia stated that the annual cost per country ranges from US$94 million to US$102 billion; of which indirect cost (monetary loss due to missed working hours, decreased productivity at work, unemployment, disability and early retirement for patients, family members, and caregivers) is 50–85%. Informal care accounts for more than 50% of this indirect cost.^3^ Another study for the US population revealed that indirect cost for schizophrenia was US$18.68 billion per year (mean patient cost = US$24664), compared with direct cost, which was US$4 billion per year (mean patient cost = US$5984).^4^

It has been observed that long-acting injectable (LAI) antipsychotics may ease the burden of daily dosing and patient compliance; however, evidence showing usefulness of LAI in alleviating caregiver burden is lacking. Since the newer LAI antipsychotic paliperidone palmitate 3-monthly (PP3M) formulation (approved in the US,^5^ and in the European Union^6^ for the maintenance treatment of schizophrenia in patients previously treated with paliperidone palmitate 1-monthly [PP1M]) requires less frequent dosing (4 times a year), this treatment is potentially useful to patients and caregivers who do not have regular access to health care or who have a history of poor treatment adherence provide stable plasma levels of drug for longer time, giving caregivers and physicians more time to intervene before relapse. Additionally, with less focus medication adherence, patients, caregivers, and physicians can potentially focus on other important aspects of the patient’s health, including psychosocial treatment, substance abuse treatment, smoking cessation, health maintenance, vocational rehabilitation etc.^3^

It is of interest to determine the impact of less frequent dosing of the antipsychotics like PP1M and PP3M on caregiver burden. The current study (largest data pool for caregiver burden) contains pooled data from two large double-blind (DB), randomized, multicenter, phase 3 studies^7, 8^ (NCT01529515 and NCT01515423) and assesses the caregiver burden in PP3M-treated and PP1M-treated patients with schizophrenia. The objectives of this post hoc analysis were to assess the demographics and baseline characteristics of caregivers in these two studies and to determine any predictors of improvement or worsening in caregiver burden following LAI (PP3M and PP1M) treatment.

## Results

### Demographics and baseline characteristics

This is one of the largest sampling of caregiver burden based upon the Involvement Evaluation Questionnaire^9–11^ (IEQ) ever collected (*n* = 1498, 27 countries). Most caregivers were parents, and the average age of caregiver was 51.5 years. A majority of caregivers spent >32 hours/week caring for the patients (Table 1).

**Table 1:** Demographics and baseline characteristics

	Study 1 *n* (%)	Study 2 *n* (%)	Combined *n* (%)
Relationship of caregiver with patient			
*N*	1147	349	1496
Mother/father	573 (50)	165 (47)	738 (49)
Spouse, partner or girl/boy friend	206 (18)	56 (16)	262 (18)
Sister and brother	148 (13)	56 (16)	204 (14)
Daughter/son	78 (7)	25 (7)	103 (7)
Other	54 (5)	12 (3)	66 (4)
Friend	46 (4)	14 (4)	60 (4)
Other relative	38 (3)	14 (4)	52 (3)
Neighbor	3 (0.3)	4 (1)	7 (0.5)
Colleague/fellow student	1 (0.1)	3 (1)	4 (0.3)
Time spent by caregivers with patient (in past 4 weeks)			
*N*	1146	350	1496
More than 32 hours/week	641 (56)	227 (65)	868 (58)
17–32 hours/week	100 (9)	43 (12)	143 (10)
9–16 hours/week	92 (8)	15 (4)	107 (7)
5–8 hours/week	112 (10)	29 (8)	141 (9)
1–4 hours/week	158 (14)	31 (9)	189 (13)
Less than 1 hour/week	43 (4)	5 (1)	48 (3)
Region caregiver distribution^a^			
*N*	1147	350	1497
Europe (Non-EU)	249 (22)	198 (57)	447 (30)
Asia	405 (35)	7 (2)	412 (28)
European Union	280 (24)	29 (8)	309 (21)
North America	123 (11)	47 (13)	170 (11)
South America	86 (8)	69 (20)	155 (10)
Australia	4 (0.4)	0 (0)	4 (0.3)
Prior LAI use at study entry^a^			
*N*	1147	350	1497
Yes	122 (11)	65 (19)	187 (12)
No	1025 (89)	285 (81)	1310 (88)
Age of caregiver (years)			
*N*	1145	350	1495
mean (SD)	51.4 (13.17)	51.8 (12.54)	51.5 (13.02)

Countries included in different regions: Asia: China, Japan, Korea, Taiwan; Australia: Australia; Europe (Non-EU): Russia, Turkey, Ukraine; European Union: Austria, Belgium, Bulgaria, Czech Republic, Germany, Spain, France, Greece, Hungary, Poland, Portugal, Romania, Slovakia; North America: Canada, USA; South America: Mexico, Colombia, Brazil, Argentina

*LAI* long acting injectable

^a^ Patient related data

### Caregiver burden at baseline

Severity of caregiver burden at open-label (OL) baseline was fair-to-moderate (mean [SD] IEQ total score: 28.5 [15.07], *n* = 1405). In terms of region (region only including Australia was not summarized due to small sample size, *n* = 4), caregiver burden was highest in South America (*n* = 148, mean [SD]: 35.7 [15.81]) followed by Asia (*n* = 377, mean [SD]: 30.3 [16.16]) at baseline. In terms of race (race groups with sample size of one are not summarized here), caregiver burden was highest in “other” race category (*n* = 56, mean [SD]: 41.6 [16.38]) followed by Asian race (*n* = 380, 30.2 [16.15]) and Black or African American race (*n* = 101, 31.0 [16.94]) at baseline. At OL baseline, caregivers whose patients had been taking LAI antipsychotics prior to study entry experienced similar burden (mean IEQ total score: 29.7 points) as those who were not taking an LAI (28.3 points).

### Caregiver burden from OL baseline to DB endpoint

**Fig. 1:**
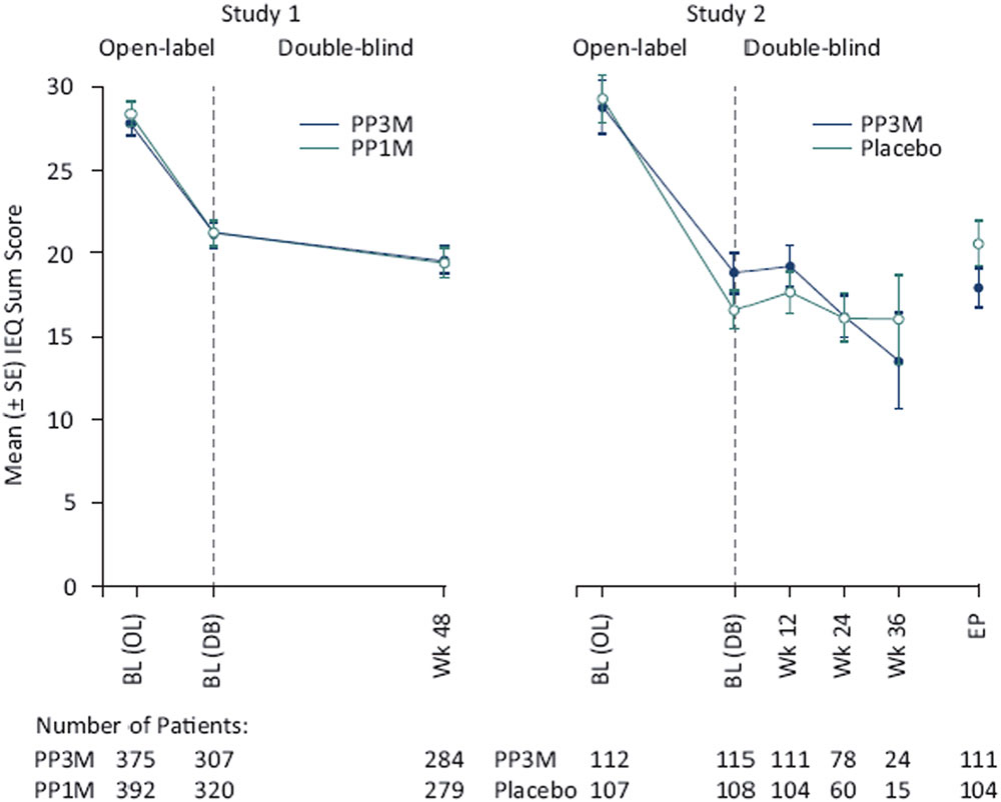
IEQ Total score over time. mITT (DB) analysis set (modified intent to treat, all patients randomized who received at least one dose of study drug during the double-blind phase and did not have any errors in the delivery of active treatment) was used for Study 1, and ITT (DB) analysis set (intent to treat, all patients who received at least one dose of study drug during DB phase) was used for Study 2. *BL* baseline, *DB* double-blind, *EP* endpoint, *IEQ* involvement evaluation questionnaire, *OL* open-label, *PP1M* paliperidone palmitate 1 month formulation, *PP3M* paliperidone palmitate 3 month formulation, *Wk* week

The overall caregiver burden gradually improved throughout the study (mean [SD] IEQ total scores change from OL baseline to DB endpoint: 8.9 [14.73] points, *n* = 756), with maximum improvements seen during the OL phase (Fig. 1). The improvements observed between the two treatment groups (PP1M and PP3M) appear similar (Fig. 1). The change in domain scores also shows a gradual improvement across time and both treatment groups. Most improvements were observed in urging domain (mean [SD] change from OL baseline to DB endpoint: −3.7 [6.45] points), followed by worrying (mean [SD] change from OL baseline to DB endpoint: −2.6 [5.11] points), tension (mean [SD] change from OL baseline to DB endpoint: −2.3 [4.84] points), and supervision (mean [SD] change from OL baseline to DB endpoint: −1.3 [3.69] points) domains.

There was a significant relationship seen between change in IEQ total scores and baseline characteristics like patient age (Table 2), patient race and region (Table 3). Region-wise, the greatest improvement between OL baseline and DB endpoint was seen in South America (*n* = 100, mean [SD]: −10.2 [16.52]) and Europe (including all European countries, regardless of European Union status, *n* = 433, mean [SD]: −9.9 [12.66]), followed by Asia (*n* = 171, mean [SD]: −6.8 [18.48]). In terms of race, the greatest improvement between OL baseline and DB endpoint was seen in “other” race (*n* = 34, mean [SD]: −16.4 [19.30]) followed by white race (*n* = 515, mean [SD]: −9.3 [12.81]). The mean (SD) improvement in Asian race (*n* = 173) and Black or African American race (*n* = 33) was −6.9 (18.42) and −5.8 (13.64), respectively.

**Table 2:** Relationship between change in IEQ total score and baseline characteristics (continuous variable)

Baseline characteristics	Estimated slope^a^	*p* value^a^
Patient age	0.106	0.0226
Patient age at diagnosis of schizophrenia	0.029	0.6211
Number of psychiatric hospitalizations within past 24 months^b^	−1.026	0.0633
Duration of latest psychiatric hospitalizations (days)	0.002	0.5371

^a^ An ANOVA model was fitted with IEQ total score change at end point (DB) from baseline (OL) as the outcome variable, and Study ID as a factor. In addition, variables for baseline characteristics were included in the model as a factor one at a time

^b^ For patients with number of hospitalization recorded as “ ≥ 4”, the value was changed to be 4 so that the model would consider “number of hospitalization” as numeric

**Table 3:** Relationship between change in IEQ total score, baseline characteristics (categorical variables) and improvement in employment status

Baseline characteristics	*N*	Change in IEQ total score Mean (SD)	*p* value^a^
Region			0.0156
Asia	171	−6.8 (18.48)	
Australia	3	−7.7 (13.43)	
Europe (non-EU)	298	−10.9 (12.86)	
European Union	135	−7.7 (11.93)	
North America	49	−4.5 (11.94)	
South America	100	−10.2 (16.52)	
Patient Race^b^			0.0156
Asian	173	−6.9 (18.42)	
Black or African American	33	−5.8 (13.64)	
Other	34	−16.4 (19.30)	
White	515	−9.3 (12.81)	
Prior LAI use at study entry			0.3947
No	663	−8.7 (14.52)	
Yes	93	−10.3 (16.17)	
Improvement in employment status			0.0516
No	688	−8.6 (14.86)	
Yes	68	−12.1 (13.08)	

*EU* European Union, *LAI* long acting injectable, *SD* standard deviation

^a^ An ANOVA model was fitted with IEQ total score change at end point (DB) from baseline (OL) as the outcome variable, and Study ID as a factor. In addition, variables for baseline characteristics (i.e., region, patient race, prior LAI use) and improvement in employment status were included in the model as a factor one at a time

^b^ Mean (SD) results for Race of “not reported” was not listed due to small sample (*n* = 1)

Patients who showed improvement in employment status had their improvement in IEQ scores more pronounced than those who did not demonstrate improvement in employment status, although the statistical testing for the association between change in IEQ scores and employment status change was not significant (*p* = 0.0516) (Table 3).

**Fig. 2:**
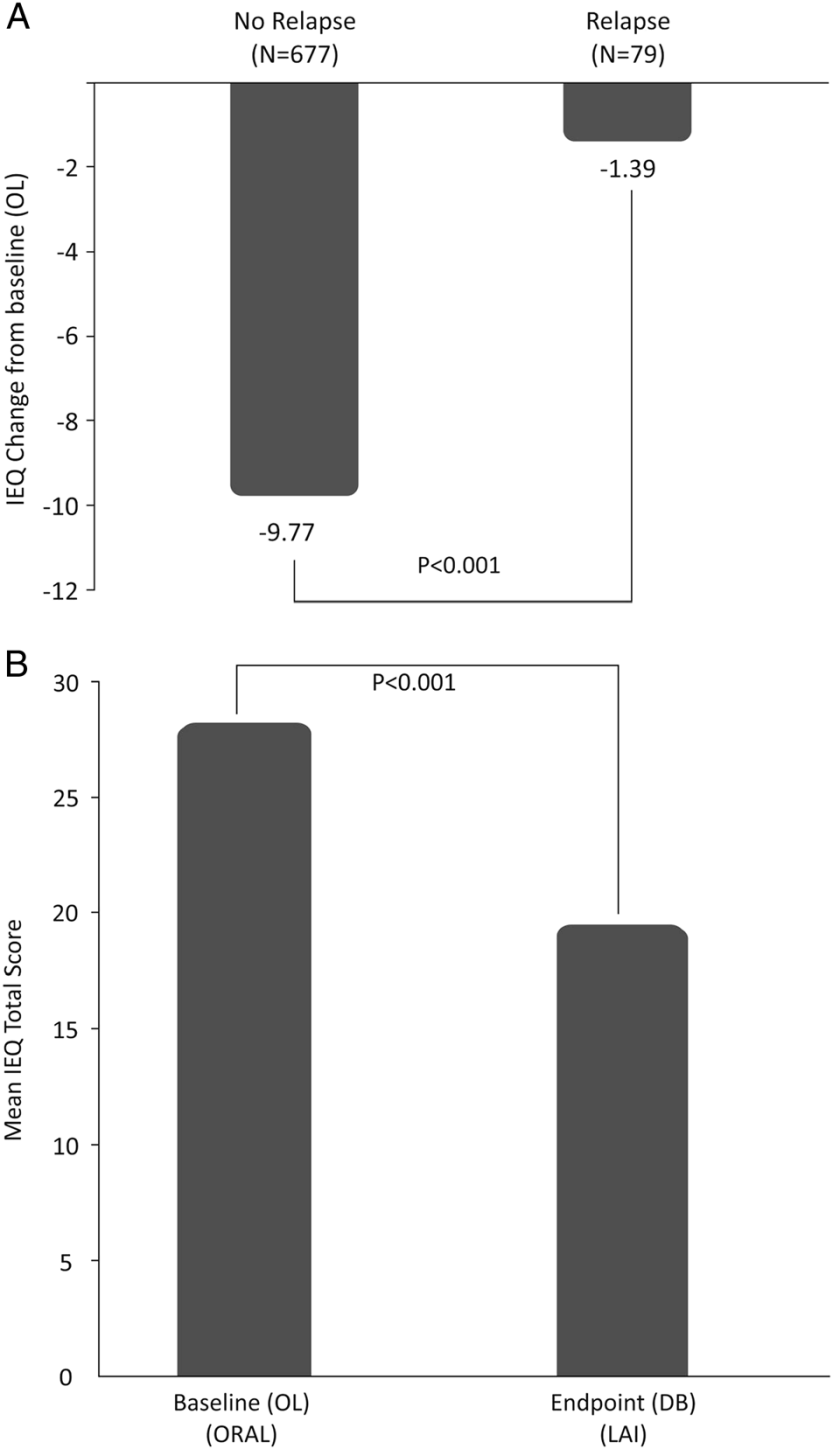
**a** IEQ Total score change at double-blind endpoint from open-label baseline, by relapse status. An ANOVA model was fitted with IEQ total score change at DB endpoint from OL baseline as outcome variable, and study ID and relapse status as factors. **b** IEQ Total score change at double-blind endpoint from open-label baseline, for patients who were on oral antipsychotics prior to study entry. A mixed model was fitted with IEQ total score as the outcome variable, and study ID and time points (OL baseline, DB endpoint) as factors. *N* = 663

Improvement in caregiver burden was significantly greater in patients without relapse (*p* < 0.001) vs. patients with relapse. Mean (SD) change in the IEQ total score from OL baseline to DB endpoint in caregivers whose relative did not relapse was −9.8 (14.59) points vs. −1.4 (13.85) points in those whose relatives had a relapse (Fig. 2a). Although there was no significant impact seen in caregiver burden from OL baseline to DB endpoint in patients with prior LAI use, as well as those with no prior LAI use, a gradual downward improvement in caregiver burden as measured by the IEQ was observed in both groups (patients with prior LAI use as well as those with no prior LAI use).

### Mirror image analysis

Before study entry, majority of patients in both studies 1 and 2 had received one or more psychotropic medications (~90% of patients).^7, 8^ The most commonly used (and ≥50%) class of psychotropic medications was the atypical antipsychotics (Study 1: 75%, Study 2: 60%) with oral risperidone being the most common atypical antipsychotic used (Study 1: 33%, Study 2: 34%).^7, 8^ For caregivers of those patients who were taking oral antipsychotics at OL baseline, after patients switching to LAI, there was a significant improvement seen in overall caregiver burden (mean IEQ total score: pre-switch [at OL baseline] 28.0 points vs post-switch [at DB endpoint] 19.3 points), number of workdays missed by caregivers over the past 4 weeks (mean number of days: pre-switch [at OL baseline] 5.4 days vs. post-switch [at DB endpoint] 2.1 days), number of leisure days of caregivers impacted over the past 4 weeks (mean number of days: pre-switch [at OL baseline] 3.4 days vs. post-switch [at DB endpoint] 1.4 days) and number of hours spent caregiving over the past 7 days (mean number of hours: pre-switch [at OL baseline] 25.6 h vs. post-switch [at DB endpoint] 18.7 h) (Table 4) (Fig. 2b).

**Table 4:** Mirror image comparison (oral antipsychotic [prior to study entry] vs LAI [During the study] use)

Outcome variable	*N*	OL baseline mean (SD)	DB end point mean (SD)	*p* value^a^
IEQ total score	663	28.0 (15.06)	19.3 (13.78)	<0.001
Number of workdays missed (past 4 weeks)	30	5.4 (5.51)	2.1 (2.63)	0.0023
Number of leisure days impacted (past 4 weeks)	654	3.4 (6.84)	1.4 (5.17)	<0.001
Number of hours spent caregiving (past 7 days)	658	25.6 (34.40)	18.7 (31.25)	<0.001

^a^ Mixed model with study ID and time points (OL baseline and DB endpoint) as factors

*IEQ* involvement evaluation questionnaire, *LAI* long acting injectable, *SD* standard deviation

## Discussion

In this study we analyzed data from two large DB, randomized, multicenter, phase 3 studies to assess the role of 2 LAI formulations of paliperidone, PP1M and PP3M, in alleviating caregiver burden in schizophrenia. Study 1 evaluated the non-inferiority of PP3M with PP1M while Study 2 was the efficacy determination trial for PP3M.^7, 8^ IEQ administered to the caregivers of patients in these studies suggested that both PP1M and PP3M formulations may alleviate the caregiver burden. Baseline characteristics show that caregiver burden (as measured by total IEQ score) was significant at the beginning of the study and improved gradually over the period of study (36–48 weeks of DB phase) though the most rapid improvement occurred during the first 17 weeks of OL treatment with PP1M. This improvement in the total IEQ score was consistent with the domain scores. Most improvement in the urging and worrying domain suggest betterment in the quality of life of caregiver.

The results of this study are of clinical relevance as caregiver burden of schizophrenia is substantial, which causes physical, emotional, and financial burdens. Yet it is underestimated by stigmatization and lack of awareness in health care professionals and society.^1^ According to a survey conducted in Ontario, Canada–half of the surveyed caregiver population ensured on daily or weekly basis that their patient took medication on time and were always concerned about it (poor treatment adherence).^12^ Symptomatic relapse in schizophrenia can be disastrous and often causes an increase in caregiver burden.^13^ Poor adherence to oral antipsychotics (irrespective of first or second generation) is major cause of symptomatic relapse in schizophrenia.^12^ Most evidence-based clinical guidelines suggest use of LAI for such patients who have history of poor adherence and relapse.^14–17^ In addition, a recent phase 3 study (NCT01515423) suggested decreased relapse rate in LAI user patients.^7^

Significant improvement in caregiver burden in patients who did not have relapse compared with population with relapse, indicate a direct relationship between core symptoms of disease or disease status (remitted or relapsed) with burden in caregivers. Prior use of LAI did not have any significance upon further improvement in the burden of caregivers. Pre and post comparison (in patients who were using oral antipsychotics before study) demonstrated alleviated overall caregiver burden and decrease in workdays missed, leisure hours impacted and number of hours spent in caregiving. All of these strongly point out towards greater potential of PP1M and PP3M towards reducing caregiver burden.

Overall, the current study represented global caregiver burden, since data was pooled from different countries (27 countries in total) and belonging to different racial and ethnic backgrounds. Both studies strongly suggested a role of formulation of antipsychotics used in the caregiver burden and benefits of LAI over conventional oral formulations. This study used a large pooled dataset for estimation of caregiver burden in schizophrenia, albeit sample size for some countries was very small. Although the two studies (from which data was pooled) were started concurrently, study 2 was terminated earlier due to positive interim analysis results (duration of study 1: 17 weeks of OL phase followed by 48 week of DB phase; duration of study 2: 17 weeks of OL transition phase, 12 weeks of OL maintenance phase and variable DB phase).^7, 8^

One of the limitations of the study is that this analysis was done post-hoc. It should also be noted that the current study did not consider the clinical stability of all patients while measuring the caregiver burden although study 2 enrolled only patients who were stable to prior antipsychotics. Schizophrenia being a chronic disease, a 48-week study may not provide a complete picture of caregiver burden and hence, longer term studies are warranted. Only non-paid caregivers were included in the study, paid caregivers were excluded, and participation was voluntary. Also, differences in caregiver burden between patients taking 1-monthly and 3-monthly LAIs could not be established in this study, as study 1 had patients receiving monthly injections regardless of assignment.

Caregiver burden was reduced significantly following PP1M and PP3M treatment (compared to the caregiver burden at the start of the study), and hence, LAI’s with their reduced frequency of administration can be an advantage. This can give caregivers more time to pay attention to other strategies of treatments such as psychosocial intervention (e.g., cognitive remediation therapy and psychoeducation) and management of side effects of drug (e.g., weight gain, sedation or extrapyramidal symptoms).^18^

## Conclusions

This post-hoc analysis showed that caregiver burden in family members of patients treated for schizophrenia is considerable. Switching from an oral antipsychotic to an LAI can provide a meaningful and significant improvement in caregiver burden.

## Methods

### Study population

Data pooled from two DB, randomized, controlled trials of PP3M were used for these analyses. Details on the study design of these 2 studies can be found elsewhere.^7, 8^ Briefly, patients with schizophrenia (DSM-IV-TR diagnosis of schizophrenia for at least 1 year before screening, total positive and negative symptom score score between 70 and 120) were first started on PP1M for 17 weeks (OL phase). In the first study, after stabilization with PP1M, patients were randomly assigned to either continue on PP1M or be switched to PP3M in the DB phase (48 weeks). The primary endpoint was proportion of patients who were relapse free at the end of 48 weeks of the DB phase (Supplementary Fig 1). In the second study, after treated with PP1M for 17 weeks, patients were treated with a single dose of PP3M for 12 weeks in the OL phase and were then randomly assigned to be dosed every 12 weeks with PP3M or be switched to placebo treatment in DB randomization phase; the primary endpoint was time to relapse. It is important to note that in the second study, patients were allowed to enter the study after having been on another oral or LAI antipsychotic.^8^ These patient data are the source of the mirror image analysis described later, which compares the burden before and after starting an LAI.

Both study protocols and their respective amendments were reviewed by an Independent Ethics Committee or Institutional Review Board for each site. These studies were conducted in compliance with the Declaration of Helsinki and applicable regulatory requirements. Written informed consent was obtained from all patients before study enrollment.

### Assessment

#### Caregiver burden evaluation

Caregiver burden was evaluated using a validated and well-accepted instrument, IEQ.^9–11^ The IEQ was chosen because it addresses a broad range of domains of caregiving consequences. It encompasses 46 total items, each rated on a scale of 0 to 4; the items are related to the encouragement and care that the caregiver has to give to the patient, to personal problems between patient and caregiver, and to the caregiver’s worries, coping and subjective burden.^9–11^ Only Module 2 (items 16–46) was included in these studies (Supplementary Table 1). Out of the 31 items on the IEQ questionnaire, 27 items were summarized into 4-distinct sub-scales: tension (9 items), supervision (6 items), worrying (6 items), urging (8 items) and a sum score of the 27 items were evaluated as a continuous variable. Item 31, “how often are you able to pursue your own activities?” was not included in the IEQ domain or total scores.

The IEQ was offered to all caregivers who participated in either study. Only those caregivers who were family members or friends who had at least 1 h of contact with the patient per week were allowed to fill out the IEQ. The same caregiver was requested to complete the questionnaire throughout the entire duration of the studies. Paid caregivers (ie caseworkers) were not permitted to complete the IEQ instrument.

### Statistical methods

Data for baseline characteristics (demographic data, prior antipsychotic [LAI vs. oral] use), relapse status, IEQ total scores, individual item scores and domain scores were pooled from the two studies. All analyses were conducted using SAS version 9.2.

ANOVA or ANCOVA models were used with change from baseline in IEQ total or domain score as the outcome variable, study ID as a factor, and variables for baseline characteristics or relapse status fitted into the model one at a time. The number of patients with improvement in occupational status were determined as those who were unemployed at OL baseline (i.e., unemployed but seeking work, unemployed but not seeking work, retired, housewife or dependent husband) and experienced a postbaseline improvement in occupational status (i.e., shift to full-time employment or gainfully self-employed, part-time employment, casual employment, sheltered work, unpaid work, student).

Caregiver burden at OL baseline and DB endpoint was compared using a mixed model, including study ID and time points (OL baseline and DB endpoint) as factors. Mirror image analysis was conducted to evaluate if caregivers experience less burden if their patients had the drug regimen switched from oral to LAI. Mirror image analysis (pre- and post-comparison) was limited only to patients who were taking an oral antipsychotic prior to study entry and who had both OL baseline and DB endpoint IEQ data. Patients on LAI antipsychotic at study entry were excluded. All LAI treatment groups were pooled together (PP1M/PP3M), including patients (from Study 2) who were randomized in DB to placebo but were exposed to PP1M/PP3M in the OL phase.

### Data Availability

The study protocols are available at http://www.nature.com/protocolexchange/

## Electronic supplementary material

Study Design

Sample Questionnaire of involvement Evaluation Questionnaire (IEQ; Items 16-46) and IEQ Domains
