# Supplementary figures and images for: Caregiver burden in schizophrenia following paliperidone palmitate long acting injectables treatment: pooled analysis of two double-blind randomized phase three studies
Source: NPJ Schizophr. 2017 Jul 27;3:23. doi: 10.1038/s41537-017-0025-5 (PMC5532271; doi:10.1038/s41537-017-0025-5)

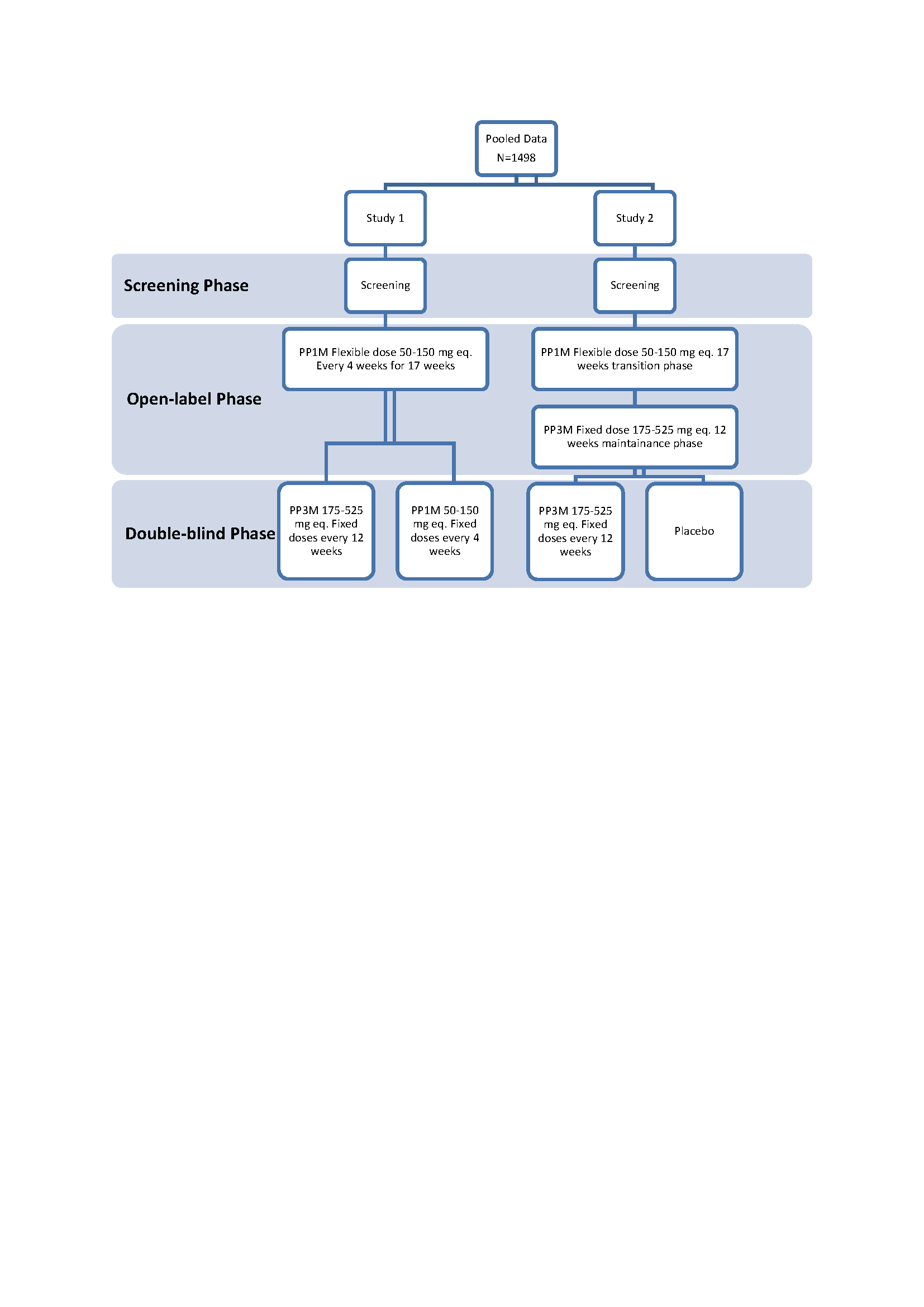

Supplement: Supplementary file 1 — Study Design [file 41537_2017_25_MOESM1_ESM.tif]
