# Supplementary material for: Caregiver burden in schizophrenia following paliperidone palmitate long acting injectables treatment: pooled analysis of two double-blind randomized phase three studies
Source: NPJ Schizophr. 2017 Jul 27;3:23. doi: 10.1038/s41537-017-0025-5 (PMC5532271; doi:10.1038/s41537-017-0025-5)
Supplement: Supplementary file 2 — Sample Questionnaire of involvement Evaluation Questionnaire (IEQ; Items 16-46) and IEQ Domains [file 41537_2017_25_MOESM2_ESM.docx]

**Supplementary** **Table 1A. Sample Questionnaire of involvement Evaluation Questionnaire (IEQ; Items 16-46)**

|  | 0 | 1 | 2 | 3 | 4 |
| --- | --- | --- | --- | --- | --- |
| Have you encouraged your relative/friend to take proper care of her/himself (e.g. bathing, brushing teeth, and dressing)? |  |  |  |  |  |
| Have you helped your relative/friend take proper care of her/himself? |  |  |  |  |  |
| Have you encouraged your relative/friend to eat enough? |  |  |  |  |  |
| Have you encouraged your relative/friend to undertake some kind of activity (e.g. go for a walk, have a chat, hobbies)? |  |  |  |  |  |
| Have you accompanied your relative/friend on some kind of outside activity, because he/she did not dare to go alone? |  |  |  |  |  |
| Have you ensured that your relative/friend has taken the required medicine? |  |  |  |  |  |

**Supplementary Table 1B: IEQ Domains**

| **IEQ Domain Score** | **No. of Items** | **Range** |
| --- | --- | --- |
| Tension | 9 | 0 (none) to 36 (extreme tension between patient and caregiver) |
| Supervision | 6 | 0 (none) to 24 (ensuring the safety of the patient requires intensive caregiver supervision) |
| Worrying | 6 | 0 (none) to 24 (patient-caregiver relationship causes extreme worry for the caregiver) |
| Urging | 8 | 0 (none) to 32 (patient requires urging by the caregiver for most daily activities and self-care) |
| Total | 27^a^ | 0 (none) to 108 (extreme caregiver burden) |

0: Never; 1: Sometimes; 2: Regularly; 3: Often; 4: (almost) Always; ^a^Because IEQ items 29 and 43 were included in more than 1 domain score, total scores were computed as the sum of the 27 items rather than as the sum of the domain scores. Item 31- “How often are you (caregiver) able to pursue your own activities?”-was not included in IEQ domain or total scores. IEQ: Involvement Evaluation Questionnaire
